# Using a simple point-prevalence survey to define appropriate antibiotic prescribing in hospitalised children across the UK

**DOI:** 10.1136/bmjopen-2016-012675

**Published:** 2016-11-03

**Authors:** Myriam Gharbi, Katja Doerholt, Stefania Vergnano, Julia Anna Bielicki, Stéphane Paulus, Esse Menson, Andrew Riordan, Hermione Lyall, Sanjay Valabh Patel, Jolanta Bernatoniene, Ann Versporten, Maggie Heginbothom, Herman Goossens, Mike Sharland

**Affiliations:** 1 NIHR Health Protection Research Unit Antimicrobial Resistance and Healthcare Associated Infection—Department of Primary Care and Public Health, Imperial College London, London, UK; 2 Paediatric Infection Diseases, St George's Hospital NHS Trust, London, UK; 3 Institute for Infection and Immunity—Paediatric Infectious Diseases Research Group, St. George's University of London, London, UK; 4 Paediatric Infectious Diseases and Immunology, Alder Hey Children's NHS Foundation Trust, Liverpool, UK; 5 Department of General Paediatrics, Evelina London Children's Hospital, London, UK; 6 Department of Infectious Diseases, St Mary's Hospital Imperial College Healthcare NHS Trust, London, UK; 7 Paediatric Infectious Diseases and Immunology, Southampton Children's Hospital, Southampton, UK; 8 Paediatric Infectious Disease and Immunology, University Hospitals Bristol NHS Foundation Trust, Bristol Royal Hospital for Children, Bristol, UK; 9 Department of Medical Microbiology, Vaccine & Infectious Disease Institute (VAXINFECTIO) University of Antwerp, Antwerp, Belgium; 10 National Public Health Service for Wales, Cardiff, UK

**Keywords:** Antimicrobials resistance, Paediatric practice, Surveillance, Quality indicators, Benchmarking

## Abstract

**Background:**

The National Health Service England, Commissioning for Quality and Innovation for Antimicrobial Resistance (CQUIN AMR) aims to reduce the total antibiotic consumption and the use of certain broad-spectrum antibiotics in secondary care. However, robust baseline antibiotic use data are lacking for hospitalised children. In this study, we aim to describe, compare and explain the prescription patterns of antibiotics within and between paediatric units in the UK and to provide a baseline for antibiotic prescribing for future improvement using CQUIN AMR guidance.

**Methods:**

We conducted a cross-sectional study using a point prevalence survey (PPS) in 61 paediatric units across the UK. The standardised study protocol from the Antibiotic Resistance and Prescribing in European Children (ARPEC) project was used. All inpatients under 18 years of age present in the participating hospital on the day of the study were included except neonates.

**Results:**

A total of 1247 (40.9%) of 3047 children hospitalised on the day of the PPS were on antibiotics. The proportion of children receiving antibiotics showed a wide variation between both district general and tertiary hospitals, with 36.4% ( 95% CI 33.4% to 39.4%) and 43.0% (95% CI 40.9% to 45.1%) of children prescribed antibiotics, respectively. About a quarter of children on antibiotic therapy received either a medical or surgical prophylaxis with parenteral administration being the main prescribed route for antibiotics (>60% of the prescriptions for both types of hospitals). General paediatrics units were surprisingly high prescribers of critical broad-spectrum antibiotics, that is, carbapenems and piperacillin-tazobactam.

**Conclusions:**

We provide a robust baseline for antibiotic prescribing in hospitalised children in relation to current national stewardship efforts in the UK. Repeated PPS with further linkage to resistance data needs to be part of the antibiotic stewardship strategy to tackle the issue of suboptimal antibiotic use in hospitalised children.

**Strengths and limitations of this study**

- We used a simple, rigorous, validated and standardised point prevalence method to provide the baseline for antimicrobial prescribing in hospitalised children to assess current and future national strategies in the UK.
- Data were collected from a large sample of hospitalised children on antibiotics (n=1247) including a wide variety of different hospitals (61 institutions) across the UK, wards and patient characteristics.
- Data were collected at the patient level providing information on the paediatric antimicrobial prescribing in secondary care adjusted on the case mix.
- Only volunteer hospitals were including in this cross-sectional study leading to potential selection biases and limited temporal relationship between antimicrobial prescribing and covariates.
- No consensus exists for measuring antibiotic prescribing in children as defined daily doses/100 inpatients is not a validated measure for this population.

## Introduction

The increasing levels of antimicrobial resistance (AMR) are strongly correlated with inappropriate use of antibiotics.^1^ ^2^ Recent UK and international reports have advocated the critical need to monitor and control the use of existing antibiotics since the number of new classes of antibiotics has dramatically decreased over the past 40 years.^3–5^ Antimicrobial stewardship programmes (ASPs), defined as comprehensive quality improvement activities for optimising antimicrobial prescribing and minimising resistance, have been widely adopted in adult care settings,^6^ ^7^ but still remain limited in children's units.^8^ ^9^ The heterogeneity in age and weight of children, as well as the lack of a standardised method to quantify antibiotic use in paediatrics, increases the challenge of determining and benchmarking the appropriateness of prescribing within or between children institutions,^10–12^ and children are often excluded from comparative studies on antibiotic use.^13^ ^14^

The National Health Service England, Commissioning for Quality and Innovation for Antimicrobial Resistance (AMR CQUIN) 2016/2017, aims to reduce by 1% or more per year the total antibiotic consumption and the use of certain broad-spectrum antibiotics considered as critical antibiotics (carbapenems and piperacillin-tazobactam) in secondary care.^15–17^ However, robust baseline antibiotic use data, so far developed for adults, are lacking for hospitalised children while they are key to measure the impact of the proposed strategies and to identify room for improvement. Two international studies have proposed to describe and compare the use of antimicrobials in children across Europe and worldwide using various quality indicators,^18^ ^19^ but no comparable detailed information on antibiotic use in hospitalised children in the UK is available.

The aim of our study is to describe, compare and explain the prescription pattern of antibiotics across paediatric units in the UK collected in a cross-sectional point prevalence survey (PPS) carried out as part of the Antibiotic Resistance and Prescribing in European Children (ARPEC) project.^20^ ^21^ We also proposed to use the simple PPS to apply AMR CQUIN quality indicators to provide a baseline of antibiotic prescribing in children to measure the impact of the current and future national strategies.

## Methods

### Study design and settings

Detailed antimicrobial prescribing data were collected for all inpatients aged under 18 years present in a participating hospital's paediatric and neonatal wards at 08:00 since at least midnight. Data collection included a wide variety of different hospitals, wards and patient characteristics to be as representative as possible of hospitalised children in the UK. Data were collected on paper forms, anonymously entered, validated and reported online through the ARPEC-PPS programme. Information on surgical prophylaxis was captured for the previous 24 hours. Antimicrobial agents were analysed in accordance with the Anatomical Therapeutic Chemical (ATC) Classification.^22^ To facilitate the data collection on underlying diagnosis (defined as a pre-existing comorbidity in addition to the diagnosis of infection for which patients are prescribed antibiotics) and reason for treatment with antibiotics, predefined lists of grouped items were used.^23^ The full method is described elsewhere by Versporten *et al*.^21^

### Data extraction

For this study, we extracted and analysed data from 61 paediatric units in the UK which participated in the ARPEC-PPS organised in March to April 2011 (feasibility survey), September to November 2011 (worldwide pilot ARPEC-PPS)^21^ and October to December 2012 (full worldwide ARPEC-PPS).^19^ All inpatients under 18 years of age admitted to a paediatric ward were included. We excluded infants on neonatal units and those on children's wards under 28 days of age. We analysed antibacterials for systemic use (ATC J01).

### Data analysis

#### Descriptive analysis

Demographic data, presence or not of an underlying chronic condition, current diagnosis, hospital-acquired infections versus community-acquired infections (CAIs), therapeutic versus prophylactic prescribing, and antibiotic type, dosing and route of administration were analysed and compared between 44 district general hospitals, which provide secondary care, and 17 tertiary referral hospitals, which provide tertiary or specialised care.

#### Metrics for measuring antibiotic use

We compared two different metrics of antibiotic prescribing within and between hospitals: (1) the proportion of children on antibiotics (prevalence rate) with 95% CIs ; (2) the defined daily doses per 100 inpatients (DDD/100 inpatients), as recommended in the AMR CQUIN.^17^ ^24^ Antibiotic consumption in grams was converted into DDD using the 2013 release of the ATC Classification.^22^ The denominator ‘inpatients’ was defined in this study as the sum of inpatients in the hospital at 08:00.

#### Quality indicators for national benchmarking between UK hospitals

We explored the different inpatient antibiotic prescribing quality indicators proposed by CQUIN NHS England for AMR.^17^

1. The total amount of antibiotics prescribed using both metrics, the proportion of children receiving antibiotics and DDD/100 inpatients in different age bands. A funnel plot was used to graphically compare antibiotic prescribing between hospitals, to adjust for different hospital sizes and to identify outliers.^25^ This takes account of the variable number of cases by institution by plotting the proportion of children on antibiotics against the sample size for each hospital using a binomial distribution and 95% CI (∼2 SD). We also displayed antibiotic prescribing in DDD/100 inpatients for each hospital, as well as the median and IQR for each age band.
2. The use of the carbapenems and the use of piperacillin-tazobactam, which are both considered critically important antibiotics against extended-spectrum β-lactamase producing Gram-negative bacteria.^3^ The proportions of children on carbapenems and piperacillin-tazobactam, as well as the amount of these drugs prescribed in DDD/100 inpatients, were monitored and compared between institutions after adjusting for hospital type (district general hospitals vs tertiary referral hospitals) and presence of underlying disease.

#### Statistical analyses

We conducted comparative analyses to determine the balance between district general hospitals and tertiary referral hospitals using tests of proportions (eg, χ^2^ analysis, Fisher’s exact test) and tests of central tendency (eg, analysis of variance, sign rank). Mean total daily doses were compared by an unpaired two-sample t-test. All p values were based on a two-tailed test with p value <0.05 for significance. Statistical analysis was performed using STATA V.12 (STATA Corp, College Station, Texas, USA).

### Ethics

The responsible UK Research Ethics Committee was approached to establish the need for a formal evaluation. Written confirmation was provided that within the UK framework a fully anonymised PPS constituted surveillance and that formal review by the Research Ethics Committee was not required.

## Results

### Patient demographics

A total of 1247 (40.9%) of 3047 surveyed UK paediatric inpatients were receiving antimicrobials. Overall, 1348 indications were recorded for 1247 inpatients with a total of 1858 antibiotic prescriptions. The median age of exposed children was 2 years (IQR=0.083–8). More than two-thirds of inpatients were recruited from tertiary care centres, and from general paediatric and paediatric surgery wards (see online supplementary table).

10.1136/bmjopen-2016-012675.supp1 Supplementary table Characteristics of paediatric hospitals across the United Kingdom (during the three one-day point prevalence surveys in 2011ߝ12)

Age differences by specialty were seen among children on antibiotics. For general paediatrics, the median age of exposed children was 2 years (IQR=0.75–6), for surgery 5 years (IQR=1.25–11), for paediatric intensive care units (PICU) 0.71 years (IQR=0.08–3), for haematology–oncology–transplant 6 years (IQR=2–11) and for other medical specialties 3 years (IQR=0.75–9).

### Total use of antibiotics

#### Proportion of children on antibiotics

Table 1 shows that the proportion of children on antibiotics and the number of prescribed antibiotics was significantly higher in tertiary hospitals (43.0%, 95% CI 40.9% to 45.1%, 40 different prescribed antibiotics) than in district general hospitals (36.4%, 95% CI 33.4% to 39.4%, 30 different prescribed antibiotics, p=0.001). About two-thirds of inpatients in intensive or specialist care wards (PICU and haematology–oncology–transplant) were prescribed antibiotics in high specialist care areas compared with about one-third in general paediatrics and surgery. Multiple antibiotics were also used more frequently in children admitted to PICU (77/145, 53.1%, 95% CI 45.0% to 61.2%) and haematology–oncology–transplant units (63/92; 68.5%, 95% CI 59.0% to 78.0%) compared with children in paediatric surgery (93/214; 43.5%, 95% CI 36.8% to 50.1%) and general paediatrics (199/554, 35.9%, 95% CI 31.9% to 39.9%).

**Table 1:** Proportion of children prescribed antibiotics in paediatric acute care settings across the UK (years 2011, 2012)

	N patients treated with antibiotic	Proportion of children on antibiotic per cent (95% CI)	N antibiotic prescriptions (total of different prescribed antibiotics)	Parenteral administration n (% of prescriptions)
District general hospitals (n=958 patients)	349	36.4 (33.4 to 39.4)	479 (30)	291 (60.8)
Tertiary referral hospitals (n=2089 patients)	898	43.0 (40.9 to 45.1)	1379 (40)	861 (62.4)
General paediatric (n=1477)	554	37.5 (35.0 to 40.0)	791 (37)	467 (59.0)
PICU (n=226)	145	64.2 (57.9 to 70.5)	228 (27)	186 (81.6)
Paediatric surgery (n=597)	214	35.8 (32.0 to 39.6)	321 (29)	223 (69.5)
Haematology–oncology–transplant (n=144)	92	63.9 (56.1 to 71.7)	156 (24)	77 (49.4)
Others (n=603)	242	40.1 (36.2 to 44.0)	362 (31)	199 (55.0)
Total (n patients=3047)	1247	40.9 (39.2 to 42.6)	1858 (41)	
	N patients treated with antibiotic (N=1247)	Proportion among total children on antibiotics per cent (95% CI)	N antibiotic prescriptions (total of different prescribed antibiotics)	Parenteral administration n (% of prescriptions)
No underlying disease	487	39.1 (34.7 to 43.4)	689 (30)	483 (70.1)
Underlying disease	760	60.9 (57.5 to 64.4)	1169 (41)	669 (57.2)
Aged <1 year	347	27.8 (23.1 to 32.6)	500 (29)	337 (67.4)
Aged 1–6 years	520	41.7 (37.5 to 46.0)	734 (31)	413 (56.3)
Aged 7–11 years	174	14.0 (8.8 to 19.1)	259 (32)	159 (61.4)
Aged >12 years	206	16.5 (11.4 to 21.5)	363 (36)	243 (66.9)
	N indications for antibiotics (N=1348)	Proportion per cent (95% CI)	N antibiotic prescriptions (total of different prescribed antibiotics)	Parenteral administration n (% of prescriptions)
Surgical infection	74	5.5 (0.3 to 10.7)	137 (15)	123 (89.8)
Surgical prophylaxis	92	6.8 (1.7 to 11.9)	123 (17)	95 (77.2)
Medical prophylaxis	233	17.3 (12.4 to 22.16)	285 (29)	25 (8.8)
Sepsis/CRBSI/CNS/febrile neutropaenia	246	18.2 (13.4 to 23.0)	385 (22)	371 (96.4)
URTI	73	5.4 (0.2 to 10.6)	90 (14)	42 (46.7)
LRTI/UTI/SSTI/joint bone/fever/GITI	569	42.2 (38.1 to 46.3)	764 (35)	458 (60.0)
Other/unknown	61	4.6 (0.0 to 9.7)	74 (22)	38 (51.4)
Community-acquired infection	797	59.1 (55.7 to 62.5)	1121 (34)	774 (69.1)
Hospital-acquired infection	211	15.7 (10.8 to 20.6)	298 (28)	240 (80.5)
Other (prophylaxis or unknown)	340	25.2 (20.6 to 29.8)	439 (34)	138 (31.4)
Total	1348		1858 (41)	

CRBSI, catheter-related bloodstream infection; CNS, central nervous system; GITI, gastrointestinal tract infection; LRTI, lower respiratory tract infection; PICU, paediatric intensive care units; SSTI, skin and soft tissue infection; URTI, upper respiratory tract infection; UTI, urinary tract infection.

Among all children receiving antibiotics, 60.9% (95% CI 57.5% to 64.4%) of children had an underlying disease compared with 39.1% (95% CI 34.7% to 43.4%) of previously healthy children. Exposed children were more likely to be younger (69.5% exposed below 7 years of age compared with 30.5% at 7 years and older).

Of 1348 indications, a diagnosis of lower respiratory, urinary tract, skin and soft tissue, bone or joint infection, fever and gastrointestinal infection was recorded in 42.2% (CI 38.1% to 46.3%) compared with 18.2% (CI 13.4% to 23.0%) with a diagnosis of severe infections, that is, sepsis, catheter-related bloodstream infection (CRBSI), central nervous system (CNS) infection or febrile neutropaenia. For exposed children, treatment for CAIs was almost four times more common (59.1%, CI 55.7% to 62.5%) than for healthcare-associated infection (15.7%, CI 10.8% to 20.6%). Finally, about a quarter of children on antibiotic therapy received either medical (17.3%) or surgical (6.8%) prophylaxis.

**Figure 1:**
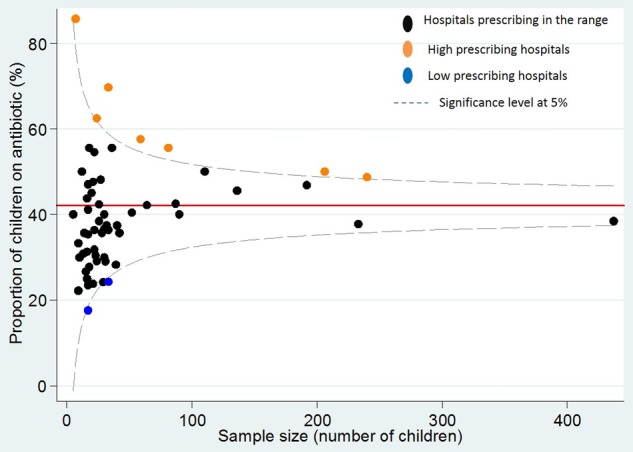
Funnel plot comparing hospital prescribing in the UK using proportion of children on antibiotics.

Figure 1 shows the funnel plot of the proportion of children on antibiotic for each institution. Hospitals with a proportion outside the funnel plot's 2 SD control limits are considered to be potential outliers. Seven of the 61 institutions were identified as potential ‘high prescribers’, 2 district general hospitals (21 children on antibiotics) and 5 tertiary referral hospitals (322 children on antibiotics). For the two district general hospitals, all children on antibiotics were from general paediatric wards, aged under 7 years for 76.2% of them (mainly aged between 1 and 6), with 52.4% of them having an underlying disease and 80.1% with a common bacterial infection (lower respiratory tract infection, urinary tract infection, skin and soft tissue infection, joint bone tissue infection). For the five tertiary hospitals, a high proportion of children on antibiotics (30.1%) were from haematology/oncology/transplant units and PICU, with a total of 71.4% of children having an underlying disease and 22.7% of them presenting with a severe infection (sepsis/CRBSI/CNS/febrile neutropaenia) while 21.1% were on medical prophylaxis. In total, 73.9% of the children were aged below 7 years (35.4%<1 and 38.5% between 1 and 6).

#### Proportion of prescriptions for parenteral versus oral administration

Parenteral was the main prescribed route for administrating antibiotics, with more than 60% of the prescriptions in district general hospitals and tertiary referral hospitals. Parenteral antibiotics were highly prescribed in PICU (81.6% of the prescriptions), for previous healthy children (70.1% of the prescriptions), for surgical infections (89.8% of the prescriptions) and for sepsis, CNS infections and febrile neutropaenia (96.4% of the prescriptions) (table 1).

#### Total usage of antibiotics in children in DDD/100 inpatients

Table 2 illustrates the total usage of antibiotics in DDD/100 inpatients for each age category per type of hospital and specialty. The total amount of antibiotics used is slightly higher in tertiary hospitals than in district general hospitals (37.8 vs 30.7 DDD/100 inpatients), except for children aged 1–6 years. The use of antibiotics is about twice as common in haematology–oncology–transplant units compared with other specialties, especially for patients aged above 12 years. For patients aged below 1 year, the use of antibiotics is substantially higher in PICU compared with other specialties.

**Table 2:** Total usage of antibiotics in DDD/100 inpatients in paediatric acute care settings across the UK, year 2011–2012

	DDD/100 inpatients			
	Aged <1 year	Aged 1–6 years	Aged 7–11 years	Aged >12 years
District general hospitals, n=958	3.2	12.3	6.0	9.2
Tertiary referral hospitals, n=2089	4.0	10.5	7.1	16.2
General paediatric, n=1477	3.9	11.9	4.7	12.8
PICU, n=226	7.5	12.7	6.4	10.9
Paediatric surgery, n=597	2.1	9.0	10.7	15.7
Haematology–oncology–transplant, n=144	0.45	14.2	14.0	31.7
Others, n=603	4.3	9.7	6.3	11.9
Total	32.9	64.8	118.3	207.5

DDD, defined daily doses; PICU, paediatric intensive care units.

**Figure 2:**
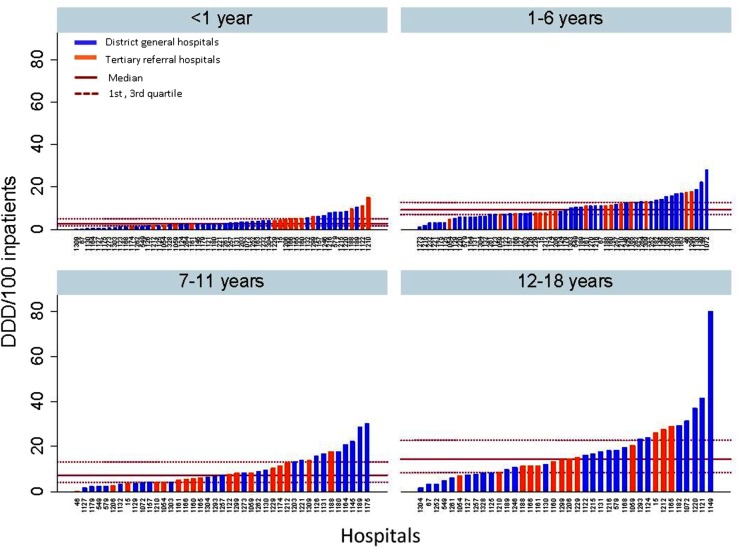
Total prescribed antibiotics (DDD/100 inpatients) per age class and type of hospital across the UK during the point prevalence survey in 2011–1012. DDD, defined daily doses.

The total prescribed antibiotics in DDD/100 inpatients per age band is shown in figure 2. A wide range of antibiotic use is observed among the 61 centres for patients aged between 12 and 18 years, whereas the three other groups show greater homogeneity between institutions in antibiotic usage. The total prescribed antibiotics are harmonised between district general hospitals and tertiary referral hospitals across the four age groups.

### Carbapenems and piperacillin-tazobactam

Table 3 shows that among children receiving at least one antibiotic, the proportion of children on carbapenems was significantly higher in tertiary hospitals than in district general hospitals (respectively, n=54, 6.0% vs n=7, 2.0%, p=0.003). The same results were observed for the total amount of DDD/100 inpatients. Less than half of the children on carbapenems had at least one underlying disease recorded for district general hospitals, while more than 9 out of 10 had an underlying disease for tertiary hospitals. In district general hospitals, the general paediatric wards were the main prescribers of carbapenems as an empirical treatment, whereas in tertiary hospitals about 43% of the prescriptions were targeted and PICU were the main prescribers.

**Table 3:** Total usage of carbapenems and piperacillin-tazobactam in paediatric acute care settings across the UK, year 2011–2012

	Carbapenems		Piperacillin-tazobactam	
	District general hospitals (349 children on antibiotics)	Tertiary referral hospitals (898 children on antibiotics)	District general hospitals (349 children on antibiotics)	Tertiary referral hospitals (898 children on antibiotics)
Total DDD/100 inpatients	36.4	56.0	39.7	20.0
Total children, n (%)*	7 (2.0)	54 (6.0)	14 (4)	68 (7.6)
General paediatric, n children (%)†	6 (85.7)	14 (25.9)	11 (78.6)	9 (13.2)
PICU, n children (%)	1 (14.3)	17 (31.5)	0	12 (17.6)
Paediatric surgery, n children (%)	0	6 (11.1)	3 (21.4)	7 (10.3)
Haematology–oncology–transplant, n children (%)	0	10 (18.5)	0	19 (27.9)
Others, n children (%)	0	7 (13.0)	0	21 (30.9)
Underlying disease vs previously healthy children, n children (%)†	3 (42.9)	49 (90.7)	12 (85.7)	67 (98.5)

*Per cent among the total number of children on antibiotics per type of hospitals.

†Per cent among the number of children on carbapenems or piperacillin-tazobactam.

DDD, defined daily doses; PICU, paediatric intensive care units.

The amount of piperacillin-tazobactam in DDD/100 inpatients was also surprisingly twofold higher in district general hospitals than in tertiary hospitals. However, the proportion of children on piperacillin-tazobactam among all the children on antibiotics was much higher in tertiary hospitals. In district general hospitals, most of the patients were prescribed piperacillin-tazobactam in paediatric general wards as an empirical treatment when they had at least one underlying disease, whereas in tertiary hospitals piperacillin-tazobactam was prescribed in haematology–oncology–transplant wards in the presence of an underlying disease.

## Discussion

We describe a unique inpatient antibiotic prescribing data set from 61 paediatric units across the UK. Our results identified areas of potential improvement for appropriate prescribing at the patient level adjusting for risk factors (age, underlying diseases, infections, specialties), using the paediatric point prevalence method developed by the ARPEC project. Our results provide the baseline for future benchmarking to monitor national strategies for optimal antimicrobial prescribing in children, particularly the CQUIN NHS England scheme 2015/2016 for AMR.

A total of 1247 out of 3047 surveyed admitted children were on antibiotics in this study. The proportion of children receiving antibiotics showed a wide variation between district general hospitals and tertiary referral hospitals, as well as a wide variation within both groups of hospitals. The presence of case mix and specialties, such as haematology–oncology–transplant and PICU, may be responsible for some of the differences observed in prescribing. Figure 1 highlighted that a total of 7/61 (11.5%) institutions, mainly the haematology–oncology–transplant and PICU units of the tertiary hospitals, were identified as potential ‘high prescribers’. However, potential ‘high prescribers’ in general district hospitals were only general paediatric units with less than half of the patients having an underlying disease.

We also highlighted a proportion of patients on medical prophylaxis (17.3%) similar to that in other countries (16.9% in Italy and 14.8% on average worldwide).^19^ ^26^ Medical prophylaxis appeared to be one of the most common indications for antibiotic prescribing in children. The reason, duration and need for prophylaxis should be further assessed for quality improvement through ASPs across paediatric units in the UK, as it is in adult settings.^27^

The total usage of antibiotics in DDD/100 inpatients per age group showed a higher consumption in haematology–oncology–transplant units compared with the other specialties, except for those aged under 1 year receiving antibiotics in PICU. Children admitted to haematology–oncology–transplant units or to PICU were more likely to receive a combination of antibiotics than general and surgical paediatric patients, which may directly impact on exposure measured in DDD/1000 inpatients.

Carbapenems and piperacillin-tazobactam were mainly prescribed empirically, and to children with underlying conditions in tertiary hospitals. These results are expected and will serve as a benchmark in future evaluations. However, we did not predict that general paediatric units were high prescribers for these two drugs in district general and tertiary hospitals. With the spread of extended-spectrum β-lactamase producing *Enterobacteriaceae* in adults^28^ as well as in paediatrics over the past decade,^29^ and the increase of small outbreaks of multidrug-resistant organisms in UK paediatric hospitals,^30^ the prescribing pattern for these critical drugs may change in the future and needs to be better monitored, especially in the general paediatric units for previously healthy children.

There remains a lack of consensus regarding the optimal metric to assess paediatric antimicrobial use, which is an important limitation. The use of DDD/100 inpatients (DDD being defined as the amount of antibiotic prescribed for a 70 kg average adult weight for its main indication) proposed by CQUIN AMR is not a perfect measure, especially in children with a wide range of weights (from 5 kg in a 3 months old to over 100 kg in obese adolescents). Since DDD is weight-dependent and dose-dependent,^31^ we decided to compare overall drug exposure using DDD/100 inpatients in age bands as proposed by Porta *et al*.^24^ Despite DDD/100 inpatients being advocated by the WHO Collaborating Centre for Drug Statistics and Methodology, ‘days of therapy’ could have advantages over DDD measures, because the impact of variation in absolute dose is limited for this metric.^11^ ^31^ However, longitudinal studies or access to electronic-prescribing systems for each hospital in the UK would be required to calculate this, which may not be realistic in the near future.^32^ For now, DDD/100 inpatients could be used to monitor changes within units over time as long as the case mix remains the same. While we have strongly promoted this study to include a large number of paediatric centres from a wide variety of different hospitals, wards and patient characteristics across the UK, only volunteer centres were recruited, with the potential for selection biases. Finally, the PPS methodology provided limited evidence on the temporal relationship between antimicrobial prescribing in children and covariates of interest.

In conclusion, we provide a robust baseline for antibiotic prescribing in hospitalised children in relation to current national stewardship efforts in the UK. Repeated PPS^33^ needs to be part of the paediatric antibiotic stewardship strategy in order to identify prescribing trends over time, to evaluate the efficacy of ASPs and to tackle the issue of suboptimal antibiotic use, especially on antibiotic dosing.^34^ International standardised PPS with further linkage between antibiotic prescribing and resistance will be critical to characterise appropriate use of antibiotics in hospitalised children globally and to propose guidance on the management of paediatric infections taking into account resistance profiles.

## References

[1] Baquero F, Negri MC, Morosini MI Antibiotic-selective environments. Clin Infect Dis 1998;27(Suppl 1):S5–11. 9710666 10.1086/514916

[2] Jacobson KL, Cohen SH, Inciardi JF The relationship between antecedent antibiotic use and resistance to extended-spectrum cephalosporins in group I beta-lactamase-producing organisms. Clin Infect Dis 1995;21:1107–13. 8589129 10.1093/clinids/21.5.1107

[3] Davies SC, Gibbens N UK five year antimicrobial resistance strategy 2013 to 2018. London: Department of Health, 2013.

[4] WHO. The evolving threat of antimicrobial resistance: options for action. Geneva: World Health Organisation, 2012.

[5] CDC. Antibiotic resistance threats in the United States. Atlanta: Centres for Disease Control and Prevention, 2013.

[6] Charani E, Holmes AH Antimicrobial stewardship programmes: the need for wider engagement. BMJ Qual Saf 2013;22:885–7. 10.1136/bmjqs-2013-002444 24046440

[7] Ashiru-Oredope D, Sharland M, Charani E Improving the quality of antibiotic prescribing in the NHS by developing a new antimicrobial stewardship programme: start smart—then focus. J Antimicrob Chemother 2012;67(Suppl 1):i51–63. 10.1093/jac/dks202 22855879

[8] Hersh AL, Beekmann SE, Polgreen PM Antimicrobial stewardship programs in pediatrics. Infect Control Hosp Epidemiol 2009;30:1211–17. 10.1086/648088 19852666

[9] Di Pentima MC, Chan S, Hossain J Benefits of a pediatric antimicrobial stewardship program at a children's hospital. Pediatrics 2011;128:1062–70. 10.1542/peds.2010-3589 22106075

[10] Gerber JS, Newland JG, Coffin SE Variability in antibiotic use at children's hospitals. Pediatrics 2010;126:1067–73. 10.1542/peds.2010-1275 21078728 PMC4677056

[11] Fridkin SK, Srinivasan A Implementing a strategy for monitoring inpatient antimicrobial use among hospitals in the United States. Clin Infect Dis 2014;58:401–6. 10.1093/cid/cit710 24162744 PMC4645276

[12] Fortin E, Fontela PS, Manges AR Measuring antimicrobial use in hospitalized patients: a systematic review of available measures applicable to paediatrics. J Antimicrob Chemother 2014;69:1447–56. 10.1093/jac/dku003 24481320

[13] Ibrahim OM, Polk RE Benchmarking antimicrobial drug use in hospitals. Expert Rev Anti Infect Ther 2012;10:445–57. 10.1586/eri.12.18 22512754

[14] Polk RE, Fox C, Mahoney A Measurement of adult antibacterial drug use in 130 US hospitals: comparison of defined daily dose and days of therapy. Clin Infect Dis 2007;44:664–70. 17278056 10.1086/511640

[15] ARHAI. *Advisory Committee on Antimicrobial Resistance and Healthcare Associated Infection (ARHAI) 4th Annual Report, February 2012—March 2013* Departement of Health, 2013:25.

[16] ARHAI. Advisory Committee on Antimicrobial Resistance and Healthcare Associated Infection—Antimicrobial stewardship: “START SMART—THEN FOCUS” Guidance. London, UK: Department of Health, 2011:27.

[17] NHS. Commissioning for Quality and Innovation (CQUIN)—Guidance for 2016/17 2016 Available from https://www.england.nhs.uk/wp-content/uploads/2016/03/cquin-guidance-16-17-v3.pdf.

[18] Amadeo B, Zarb P, Muller A European Surveillance of Antibiotic Consumption (ESAC) point prevalence survey 2008: paediatric antimicrobial prescribing in 32 hospitals of 21 European countries. J Antimicrob Chemother 2010;65:2247–52. 10.1093/jac/dkq309 20713405

[19] Versporten A, Bielicki J, Drapier N The Worldwide Antibiotic Resistance and Prescribing in European Children (ARPEC) point prevalence survey: developing hospital-quality indicators of antibiotic prescribing for children. J Antimicrob Chemother 2016;71:1106–17. 10.1093/jac/dkv418 26747104

[20] Henderson KL, Muller-Pebody B, Johnson AP First set-up meeting for Antibiotic Resistance and Prescribing in European Children (ARPEC). Euro Surveill 2009;14:pii: 19404 . 10.2807/ese.14.45.19404-en 19941784

[21] Versporten A, Sharland M, Bielicki J The antibiotic resistance and prescribing in European Children project: a neonatal and pediatric antimicrobial web-based point prevalence survey in 73 hospitals worldwide. Pediatr Infect Dis J 2013;32:e242–53. 10.1097/INF.0b013e318286c612 23838740

[22] WHO. Guidelines for ATC classification and DDD assignment 2013. Oslo: WHO Collaborating Centre for Drug Statistics Methodology, 2012.

[23] Porta A, Esposito S, Menson E Off-label antibiotic use in children in three European countries. Eur J Clin Pharmacol 2010;66:919–27. 10.1007/s00228-010-0842-1 20532493

[24] Porta A, Hsia Y, Doerholt K Comparing neonatal and paediatric antibiotic prescribing between hospitals: a new algorithm to help international benchmarking. J Antimicrob Chemother 2012;67:1278–86. 10.1093/jac/dks021 22378680

[25] Spiegelhalter DJ Funnel plots for comparing institutional performance. Stat Med 2005;24:1185–202. 10.1002/sim.1970 15568194

[26] De Luca M, Dona D, Montagnani C Antibiotic prescriptions and prophylaxis in Italian children. is it time to change? Data from the ARPEC Project. PLoS ONE 2016;11:e0154662. 27182926 10.1371/journal.pone.0154662 PMC4868290

[27] Bruce J, MacKenzie FM, Cookson B Antibiotic stewardship and consumption: findings from a pan-European hospital study. J Antimicrob Chemother 2009;64:853–60. 10.1093/jac/dkp268 19675012

[28] ECDC. European Centre for Disease Prevention and Control (ECDC)- EARS-net database. Secondary European Centre for Disease Prevention and Control (ECDC)- EARS-net database 2015 http://www.ecdc.europa.eu/en/healthtopics/antimicrobial_resistance/database/Pages/database.aspx

[29] Lukac PJ, Bonomo RA, Logan LK Extended-spectrum beta-lactamase-producing Enterobacteriaceae in children: old foe, emerging threat. Clin Infect Dis 2015;60:1389–97. 10.1093/cid/civ020 25595742 PMC4415054

[30] Drew RJ, Turton JF, Hill RL Emergence of carbapenem-resistant Enterobacteriaceae in a UK paediatric hospital. J Hosp Infect 2013;84:300–4. 10.1016/j.jhin.2013.05.003 23831281

[31] Gravatt LA, Pakyz AL Challenges in measuring antibiotic consumption. Curr Infect Dis Rep 2013;15:559–63. 10.1007/s11908-013-0374-9 24097249

[32] Ahmed Z, McLeod MC, Barber N The use and functionality of electronic prescribing systems in English acute NHS trusts: a cross-sectional survey. PLoS ONE 2013;8:e80378 10.1371/journal.pone.0080378 24278279 PMC3835329

[33] Willemsen I, Groenhuijzen A, Bogaers D Appropriateness of antimicrobial therapy measured by repeated prevalence surveys. Antimicrob Agents Chemother 2007;51:864–7. 10.1128/AAC.00994-06 17210766 PMC1803106

[34] Lestner JM, Versporten A, Doerholt K Systemic antifungal prescribing in neonates and children: outcomes from the Antibiotic Resistance and Prescribing in European Children (ARPEC) Study. Antimicrob Agents Chemother 2015;59:782–9. 10.1128/AAC.04109-14 25403672 PMC4335832

