# Supplementary material for: Using a simple point-prevalence survey to define appropriate antibiotic prescribing in hospitalised children across the UK
Source: BMJ Open. 2016 Nov 3;6(11):e012675. doi: 10.1136/bmjopen-2016-012675 (PMC5129034; doi:10.1136/bmjopen-2016-012675)
Supplement: Supplementary table — Characteristics of paediatric hospitals across the United Kingdom (during the three one-day point prevalence surveys in 2011ߝ12) [file bmjopen-2016-012675supp_table.pdf]

Supplemental table: Characteristics of paediatric hospitals across the United Kingdom (during the three one-day point prevalence surveys in 2011-12)

	N patients (%)	N beds	Bed occupancy
District general hospitals (44 centres)	958 (31.4)	1604	59.7%
Tertiary referral hospitals (17 centres)	2089 (68.6)	2542	82.2%
General Paediatric	1477 (48.5)	2235	66.1%
PICU - Paediatric Intensive Care Unit	226 (7.4)	265	85.3%
Paediatric Surgery	597 (19.6)	789	75.7%
Haematology-oncology-transplant	144 (4.7)	195	73.8%
Others	603 (19.8)	662	91.1%
**Total** (N centres = 61)	3047	4146	73.5%
